# The Association Between Frailty and a Nurse-Identified Need for Comprehensive Geriatric Assessment Referral from the Emergency Department

**DOI:** 10.1177/08445621221144667

**Published:** 2023-01-11

**Authors:** Fabrice I. Mowbray, Brittany Ellis, Connie Schumacher, George Heckman, Kerstin de Wit, Ryan P. Strum, Aaron Jones, Rebecca H. Correia, Eric Mercier, Andrew P. Costa

**Affiliations:** 1 Department of Health Research Methods, Evidence and Impact, 3710 McMaster University, Hamilton, Ontario, Canada; 2 Department of Emergency Medicine, College of Medicine, 7235 University of Saskatchewan, Saskatoon, Saskatchewan, Canada; 3 School of Nursing, Faculty of Applied Health Sciences, 7497 Brock University, St. Catherine's, Ontario, Canada; 4 Department of Emergency Medicine, 4257 Queen's University, Kingston, Ontario, Canada; 5 Division of Emergency Medicine, Department of Medicine, 3710 McMaster University, Hamilton, Ontario, Canada; 6 School of Public Health and Health Systems, 8430 University of Waterloo, Waterloo, Ontario, Canada; 7 Centre de recherche du CHU de Québec, Université Laval, Québec City, Québec, Canada; 8 Centre de recherche sur les soins et les services de première ligne, Université Laval, Québec City, Québec, Canada

**Keywords:** frailty, geriatric syndromes, emergency nursing, comprehensive geriatric assessment

## Abstract

**Background:**

Emergency nurses commonly conduct geriatric assessments in the emergency department (ED). However, little is known about what geriatric syndromes or clinical presentations prompt a nurse to document an identified need for comprehensive geriatric assessment (CGA).

**Objectives:**

To examine the association between geriatric syndromes, like frailty, and a nurse-identified need for a CGA following emergency care.

**Methods:**

We conducted a secondary analysis of a multi-province Canadian cohort from the InterRAI Multinational Cohort Study. We collected data at ED registration from patients 75 years of age and older (n = 2,274) from eight ED sites across Canada between November 2009 and April 2012. Geriatric syndromes were assessed by trained emergency nurses using the interRAI ED Contact Assessment; and we retrospectively calculated the ED frailty index. We employed binary logistic regression to determine the adjusted associations between geriatric syndromes and a nurse-identified need for a CGA.

**Results:**

Approximately one-quarter (28%) of older adults were identified to need a CGA following emergency care. A 0.1 unit increase in the ED frailty index increased the likelihood of a nurse identify a need for CGA (RD: 6.6; 95% CI = 5.5–7.9). Most geriatric syndromes increased the probability of a nurse documenting the need for a CGA.

**Conclusion:**

When assessed by emergency nurses, the identified need for CGA is strongly linked to the presence of geriatric syndromes, including frailty. We provide face validity for the continued use of emergency nurses for screening and assessing older ED patients.

## Background and Purpose

Geriatric syndromes, like frailty, are robust predictors of patient-important health outcomes and health service use in older emergency department (ED) patients (Costa et al., 2014; Inouye et al., 2007; Mowbray et al., 2020a). Geriatric ED guidelines and experts have emphasized the need to evaluate and communicate geriatric syndromes and related patient vulnerabilities, both in the ED and during transitions of care (American College of Emergency Physicians et al., 2014; Carpenter & Mooijaart, 2020; Ellis et al., 2022; Perry et al., 2018). Foreknowledge of geriatric syndromes and complexity can be used to inform patient-centred care, disposition decision-making, and the need for a comprehensive geriatric assessment (CGA) both in-hospital or post-discharge (American Geriatrics Society Expert Panel on the Care of Older Adults with Multimorbidity, 2012; Perry et al., 2018).

The CGA is the gold standard approach for evaluating older adults and is defined as a multi-dimensional and systematic process to evaluate older adults’ medical, psychosocial, and functional capacities (Parker et al., 2018; Stuck et al., 1993). Older adults who receive a CGA in-hospital or following discharge have lower odds of mortality and admission to long-term care (Deschodt et al., 2013; Elkan et al., 2001; Ellis et al., 2017). In primary and community care, providing a CGA can decrease hospital visitation and admission lengths of stay in medically complex older adults (Nord et al., 2021). As the main portal of entry into local hospital systems, the ED is uniquely situated to initiate a referral for a CGA. Geriatric models of care and emergency management pathways have been slow to respond to the many calls to action and guidelines developed (Carpenter & Mooijaart, 2020; Hogan et al., 2014), and less than 5% of EDs in North America have received accreditation for geriatric care (Kennedy et al., 2022)

Geriatric services and assessment in the ED are most commonly conducted by registered nurses, ideally with specialty training in geriatric emergency management (Leaker et al., 2020; Sinha et al., 2011). Time pressures, high medical acuity, and limited geriatric expertise in the ED commonly hinder opportunities to conduct a CGA (Carpenter & Mooijaart, 2020). As a result, geriatric screeners and assessments are brief and purposed to identify the need for CGA following emergency care (Carpenter & Mooijaart, 2020; Elliott et al., 2017). Despite consensus on the importance of referring patients for specialized geriatric services from the ED (Parker et al., 2017), little is known about what geriatric syndromes or clinical presentations prompt a nurse or healthcare provider to document an identified need for CGA.

The CGA is known to be more beneficial for older adults with frailty (Ekdahl et al., 2015). Thus, our primary objective of this study was to examine the association between frailty and a nurse-identified need for a CGA following emergency care in older ED patients after accounting for triage acuity, formal support needs (e.g., home care), and hospitalization. Mindful that frailty indices and measures are traditionally scored based on the accumulation or presence of geriatric syndromes (e.g., function and cognition), we set out to delineate what distinct geriatric syndromes are associated with a nurse-identified need for a CGA as our secondary objective.

## Methods and Procedures

We conducted a secondary data analysis of the Canadian cohort from the interRAI multinational ED study (Costa et al., 2014). The Strengthening the Reporting of Observational Studies in Epidemiology (STROBE) statement was used to guide the reporting of this study (Vandenbroucke et al., 2014). Data were collected on 2,274 older adults from eight ED sites across five provinces in Canada (Ontario, Nova Scotia, Manitoba, Saskatchewan, and British Columbia). Patients aged 75 and older who presented to ED between November 2009 and April 2012 were screened consecutively for study eligibility at emergency registration. Patients were excluded if (i) they were expected to die within 24 h of emergency presentation, (ii) they presented in severe medical distress as determined by a triage nurse, or (iii) they did not speak English or French and were without an interpreter. We also excluded patients transferred from post-acute care (i.e., complex-continuing care) as this represents a population receiving extensive facility-based care from a hospital or rehabilitation center. Ethics approval for secondary analysis was obtained from the academic institutions and research ethics boards of all participating hospitals (Costa et al., 2014). A waiver of informed consent was granted, which allowed for the recruitment of older ED patients who are often excluded from clinical research, like those with cognitive impairment (Herrera et al., 2010; Watts, 2012).

### Measurement and Variables

All patients were evaluated using the interRAI ED Contact Assessment (ED-CA) upon enrollment into the study. The ED-CA is a brief assessment and a standardized clinical decision support tool to inform emergency management, discharge planning, and referral decision-making in the ED (Costa et al., 2017). The ED-CA has clinical items that assess patient condition, performance, and capacity across various domains, including physical function, cognition, comprehension, mood, falls, nutritional risk, pain, and dyspnea (Costa et al., 2017). The items of the ED-CA have established test content validity in acute care (Wellens et al., 2011), have high inter-rater reliability (Hirdes et al., 2008; Wellens et al., 2012), and high predictive validity across a series of outcomes in the ED setting (Brousseau et al., 2018; Costa et al., 2014; Mowbray et al., 2020b). The ED-CA has yet to receive a revision since its original development. To provide a pragmatic lens, geriatric assessments were conducted and documented by hospital-staffed emergency nurses who received additional standardized training on the ED-CA and supplementary software.

We measured frailty using the ED frailty index (ED-FI), an internationally validated measure for the ED setting (Brousseau et al., 2018). The ED-FI is a cumulative health deficit model presented as a fraction with the numerator as the number of health deficits present and the denominator as the total number of deficits possible in the ED-CA (Brousseau et al., 2018). We calculated frailty in a post-hoc manner using assessment data from the ED-CA. Frailty data were unavailable for decision-making during ED management or discharge, allowing for an uncontaminated view of referral recommendations.

We measured age as a continuous variable and sex as a dichotomous variable based on biological sex (i.e., male, female). We defined caregiver distress as the presence of stress, anger, or depression in the primary caregiver or support persons. We defined impaired cognitive status as patients who presented with: an acute change in mental status (e.g., restlessness, lethargy), delusions or hallucinations, difficulty comprehending verbal information most of the time, or difficulty with daily decision-making (e.g., finances, meals).

We categorized patients as (i) independent with activities of daily living (ADL), or (ii) requiring assistance or supervision with bathing, personal hygiene (i.e., brushing teeth, combing hair), dressing the lower body, or ambulation. In regards to instrumental ADLs (I-ADLs), we classified patients as (i) independent or (ii) requiring assistance or supervision with stair negotiation or medication management (administration or dosing).

We grouped patients who presented with a traumatic injury (e.g., fracture) or reported a fall in the 90 days before ED presentation. We defined nutritional risk as (i) a noticeable decrease in the amount of food or fluid consumed in the last three days, or (ii) weight loss of 5% in the 30 days, or 10% in the 180 days, prior to the index ED visit. Finally, patients reporting symptoms associated with depression or anxiety were categorized as exhibiting symptoms of a mood disorder.

In Canada, triage acuity is determined using the Canadian Triage Acuity Scale (CTAS), a five-item ordinal scale ranging from one to five, with lower numbers indicating higher medical acuity. We grouped patients with a CTAS score of one (resuscitation), two (emergent), and three (urgent) and classified them as high acuity (Bullard et al., 2014). We grouped patients who received a score of four (less-urgent) and five (non-urgent) and classified this cohort as low acuity.

### Outcome Measurement

The outcome for our study was a nurse-identified need for a CGA following emergency care. The need for a CGA was determined and documented by the assessing emergency nurse, leveraging their clinical judgement, immediately following completion of clinical items of the ED-CA. Specifically, assessors were asked “Is there a need for a comprehensive geriatric assessment?” at the time of completion. Assessors could select from one of two responses, *yes* or *no*. The referral recommendation was then communicated to the treating ED physician responsible for CGA referrals.

### Analysis

We report descriptive statistics using general measures of frequency and central tendency. We employed a multivariable logistic regression model to determine if an association exists between frailty, measured with the ED-FI, and a nurse-identified need for a CGA. We also used logistic regression to determine the adjusted associations between distinct geriatric syndromes, excluding frailty, and a nurse-identified need for a CGA. Frailty was analyzed independently from other geriatric syndromes to avoid contamination and multicollinearity. Specifically, geriatric syndromes and conditions identified in the ED-CA are used to calculate the ED-FI score.

We selected predictors *a priori* based on clinical judgement and prior work examining geriatric syndromes in the ED (Brousseau et al., 2018; Costa et al., 2014; Gray et al., 2013; Mowbray et al., 2020b). Model accuracy and precision were determined using 10-fold cross-validation and reported as a concordance statistic with 95% confidence intervals. An event-per-variable (EPV) ratio of greater than 20 was maintained for both multivariable models (Steyerberg, 2019). We deleted cases with missing data within each analysis. We managed and analyzed data using R version 4.0, and discriminative accuracy was determined using the “*pROC*” package.

## Results

This cohort contained 2,274 older adults who presented to the ED for medical attention. Half of the cohort was admitted for in-patient care from the ED. Overall, missing data was minimal, with less than one percent missing. As a result, ED-FI scores were calculated for only 89% of patients (n = 2,024). The median index score was 0.24 (range = 0–0.84), indicative of mild frailty (Rockwood et al., 2005).

A comprehensive list of patient characteristics and geriatric syndromes are displayed in Table 1*.* The mean age of the sample was 82.5 years, and most patients (61%) were female. One-fifth of older adults were assigned a low acuity triage score (CTAS 4 or 5). Cognitive impairment was present in 24% of the cohort. In our sample, 639 patients (28%; 95% CI = 26.4–30) had documentation from an emergency nurse of an identified need for a CGA. Five percent (n = 107) of the cohort was referred to geriatric services by the treating physician.

**Table 1.** Patient characteristics and geriatric syndromes (N = 2,274).

Variable	N (%)
Age^a^	82.5 (7.4)
Sex (Female)	1377 (61.4)
Caregiver Distress^b^	425 (18.7)
Cognitive Impairment^c^	534 (23.7)
Potential Delirium^d^	332 (14.7)
Activities of Daily Living Impairment	
Bathing	1318 (59.1)
Personal Hygiene	635 (28.1)
Dressing Lower Body	1007 (44.5)
Locomotion	970 (43.2)
Independent Activities of Daily Living	
Difficulty with Medications^e^	722 (31.8)
Difficulty with Stairs^f^	1379 (61)
Impaired Comprehension^g^	108 (4.7)
Conditions and Symptoms	
Poor Self-Reported Health^h^	441 (19.4)
Symptoms of depression or anxiety^i^	1005 (44.4)
Hallucinations or Delusions	145 (6.4)
Any Falls in the last 90 days	728 (32.5)
Traumatic Injury	160 (7.3)
Daily and Severe Pain^j^	421 (18.5)
Dyspnea^k^	632 (27.8)
Decrease Food Intake or Weight Loss^l^	712 (31.6)
ED Visitation Prior 90 Days	930 (40.9)
High-Risk Triage Acuity (CTAS ≤ 3)	1762 (80)
ED Frailty Index^m,n^	0.24 (0–0.84)

ADL = activities of daily living; CTAS = Canadian Triage Acuity Scale; IADL = impaired activities of daily living.

^a^ Data reported as mean (standard deviation).

^b^ Primary informal helper(s) expresses feelings of distress, anger or depression

^c^ Modified independent or any impairment in making decisions regarding tasks of daily living.

^d^ Acute change in mental status from person's usual functioning (e.g., restlessness, lethargy, difficult to arouse, altered environmental perception).

^e^ Difficulty remembering to take medicines, opening bottles, taking correct drug dosages, giving injections or applying ointments.

^f^ Supervision or the need for any assistance while walking a full flight of stairs (12 to 14 stairs).

^g^ Sometimes, rarely or never understands direct communication.

^h^ When asked, “In general, how would you rate your health?” person responds “Poor.”

^i^ When asked, patient reports feeling sad, depressed, hopeless or anxious

^j^ Pain that is excruciating or daily in past 3 days.

^k^ Dyspnea at rest or present when performing normal day-to-day activities.

^l^ Noticeable decrease in the amount of food usually eaten or fluids usually consumed OR weight loss of 5% or more in the last 30 days, or 10% or more in the last 180 days.

^m^ Measured using the Emergency Department Frailty Index (FI-ED).

^n^ Reported as median (range).

### Geriatric syndromes

Table 2 displays the results of the logistic regression model, examining the association between frailty and the identified need for a CGA. After statistically adjusting for triage acuity, admission status, and the need for formal support services (e.g., home care, long-term care), a 0.1 unit increase (10%) in the ED-FI increased the likelihood of an emergency nursing documenting an identified need for a CGA by 34%; this translates to an absolute difference of 6.6% (95% *CI* = 5.5–7.9) (Foroutan et al., 2020)**.**

**Table 2.** Association between frailty and a nurse-identified need for a CGA (N = 1,953).

Variable	aOR (95% CI)
Intercept	0.18 (0.13–0.24)
Frailty^a,b^	1.34 (1.25–1.43)
Hospital Admission	1.28 (1.04–1.59)
High-Risk Triage Acuity^c^	0.52 (0.41–0.67)
Formal support services	
Home care	3.07 (2.35–4.02)
Long-term care	0.94 (0.6–1.5)
No formal support services	–

aOR = adjusted odds ratio; CI = confidence interval;

^a^ Measured using the emergency department frailty index (ED-FI).

^b^ Reports the odds of geriatric referral per 0.1 unit increase in the FI-ED score.

^c^ Measured using the Canadian Triage Acuity Scale (CTAS); high-risk defined as CTAS 1–3.

Table 3 displays the associations between geriatric syndromes, excluding frailty, and a nurse-identified need for a CGA. The following geriatric syndromes were found to increase the odds of an identified need for a CGA: living alone, caregiver distress, impaired daily decision-making, assistance with I-ADLs, a history of falls or a current presentation of a traumatic injury, and symptoms indicative of depression or anxiety. Age, sex, ADL impairment and nutritional risk were determined to be statistically insignificant. Our model's discriminative ability was fair (AUC = 0.76; 95% CI = 0.74–0.78).

**Table 3.** Association between geriatric syndromes and a nurse-identified need for CGA (N = 2,110).

Variable	aOR (95% CI)
Intercept	0.06 (0.04–0.08)
Sex (Female)	0.88 (0.69–1.1)
Lives Alone (i.e., isolation)	1.74 (1.37–2.21)
Caregiver Distress	1.59 (1.21–2.1)
Impaired Decision-Making	1.61 (1.25–2.08)
Assistance Needed for ADLs	1.28 (0.95–1.7)
Assistance Needed for Instrumental ADLs	2.31 (1.72–3.12)
Prior Fall or Presenting Traumatic Injury	1.78 (1.41–2.23)
Nutritional Risk	0.84 (0.66–1.07)
Symptoms of a Mood Disorder Reported	1.57 (1.21–2.03)
Formal support services	
Home care	2.45 (1.86–3.23)
Long-term care	0.9 (0.56–1.43)
No formal support services	–
Hospital Admission	1.5 (1.16–1.9)
High-Risk Triage Acuity^a^	0.5 (0.38–0.66)

ADL = activities of daily living; aOR = adjusted odds ratio; CI = confidence interval; ED = emergency department.

^a^ High risk defined as CTAS score of one to three.

In both multivariable models (with and without frailty), active enrollment in home care services and the need for hospital admission increased the odds of a nurse documenting a need for CGA. Similarly, triage acuity was inversely related to the identified need for a CGA in both models; older adults with high medical acuity (CTAS 1–3) had 50% lower odds of a nurse-identified need for CGA. Both multivariable models had fair discriminative ability (AUC = 72–76).

## Discussion

Our study demonstrated a strong relationship between frailty and a nurse-identified need for a CGA following emergency care. Specifically, a 0.1 unit increase in the ED-FI corresponds with an absolute increase (6.6%) in the likelihood of a nurse identifying a need for CGA. Almost all geriatric syndromes are significantly associated with an identified need for a CGA in older ED patients. Geriatric syndromes, like frailty, are strongly associated with patient-important health outcomes in older ED patients (Costa et al., 2014; Mowbray et al., 2020a; Mowbray et al., 2020b). They also constitute the majority of assessment items within ED vulnerability screeners and the CGA (Costa et al., 2017; de Gelder et al., 2016; McCusker et al., 1999; Parker et al., 2018), highlighting their informational value and utility when determining the need for further geriatric assessment.

Our study builds on the prior work of Brousseau and colleagues who provide a crude estimate of the association between frailty and the identified need for a CGA in a similar cohort (Brousseau et al., 2018). The STROBE guidelines recommend multivariable analyses were the risk of confounding is possible (Vandenbroucke et al., 2014). Our analysis found that adjusting for triage acuity, hospitalization, and the need for formal support services decreased the relative estimate by 17%, highlighting the importance of considering these confounders in future work. Another factor to consider is the inclusion of nursing home residents in our study, a cohort excluded by the preceding study (Brousseau et al., 2018).

Over one-quarter of older ED patients were identified to need a CGA, underscoring the value of geriatric ED models of care and an opportunity to instigate an informed referral from the ED. However, only five percent received a referral for geriatric services from the treating physician, highlighting an underserved population of acutely ill older adults returning to the community. Lack of referral could be the downstream effect of a scarcity of geriatricians and geriatric-specific services in the community (Holveck & Wick, 2018). Further research is needed to understand this phenomenon and the factors that inhibit referral.

Our study protocols paralleled common practices in Canada, which require a physician or advanced practice provider's (e.g., nurse practitioner or physician assistant) assessment and signature for referral for CGA. This practice may be redundant in EDs that utilize a geriatric-trained emergency nurse to assess and refer high-risk older adults, a cost-efficient and common model of care spreading across North America (Leaker et al., 2020; Sinha et al., 2011). In our study, geriatric assessments were conducted by hospital-staffed emergency nurses, showcasing the feasibility of geriatric assessment in an emergency setting. Our findings provide preliminary data to suggest that referral for CGA could fall within the scope of nursing. Our study also provides face validity for the clinical-decision making of ED nurses regarding the need for CGA, as evidenced by the strong association between referral recommendations and geriatric syndromes, like frailty. Its also worth noting that the influential factors and decision-making of ED staff regarding the need for a CGA closely mirrored the Assessment Urgently Algorithm (AUA), an algorithm embedded within the ED-CA to score and prioritize the need for additional geriatric assessment (Costa et al., 2017).

Difficulty with I-ADLS (stairs or medication management) increased the odds of an assessor identifying a need for a CGA two-fold. However, the need for supervision or assistance with ADLs did not significantly influence the odds of an identified need for CGA. Both stair negotiation and medication management require higher levels of cognitive, motor, and sensory control when compared to ADLs (Beckman et al., 2005; Jacobs, 2016), and an inability to navigate these tasks pose a greater risk for adverse events (Hartholt et al., 2019). For example, difficulties with self-medication tasks can lead to sub-therapeutic or toxic pharmacological levels (Godfrey et al., 2013). Self-sufficiency in taking medications and navigating stairs may be perceived as critical indicators warranting further assessment via CGA following emergency care.

Nutritional risk, defined as decreased nutritional intake or recent weight loss, was another geriatric syndrome not found to prompt an identified need for a CGA. This finding is concerning, though it parallels prior work highlighting that healthcare providers report difficulty identifying nutritional risk (Duerksen et al., 2015, 2016). Nutritional status is reported to worsen in patients admitted to the hospital (Gout et al., 2009), underscoring the importance of early nutritional screening, intervention, and referral from the ED. Overlooking the identified need for CGA in patients with nutritional risk may be attributed to the fact that these patients are referred to dietary rather than geriatric services, though dietetic consults are often lacking in patients who are suffering from malnutrition (Gout et al., 2009). Nutritional risk in an older patient may indicate an array of contributors warranting CGA and dietetic assessment. Further research is needed to understand the decision-making process.

The strong relationship between frailty and the identified need for a CGA suggests that referral to specialized geriatric services may serve as a practice-based process measure, and an outcome measure, given the short lengths of stay and lack of follow-up. Administrative and health service outcomes are commonly used to determine the quality of care for older ED patients. However, these metrics are used out of convenience rather than importance. An informed assessment to identify the need for a CGA may be a more meaningful and accurate metric to evaluate the quality of ED-based geriatric assessment and referral practices.

### Strengths & Limitations

Our study used data with a comprehensive set of functional and geriatric syndromes not typically available in ED medical records. However, the secondary nature of the study limited our analyses to only those available in the archived data. Diagnostic data would have provided supplementary information to assist in understanding the factors and geriatric syndromes that prompt an identified need for a CGA from an ED clinician. Next, our data lacked information to verify the accuracy of the need identified CGA. Future studies should aim to incorporate a gold-standard reference (i.e., geriatrician assessment) to further validate the accuracy of clinical-decision making about the need for a CGA. Data were mostly collected during daytime hours; patient and visit characteristics may differ during nighttime visitation. Additionally, the data were collected approximately a decade ago, though geriatric ED models of care have been slow to adapt in recent years, increasing the generalizability of findings. Patients receiving post-acute care (i.e., complex-continuing care) were not flagged in this study. Finally, it is possible that allied health professionals conducted a handful of assessments when medical acuity and complexity were high in the ED. However, the low incidence gives confidence to generalizations made about nursing practice.

## Conclusion

Our study demonstrates that frailty, among other geriatric syndromes, is significantly associated with a nurse-identified need for a CGA in older ED patients. However, our findings highlighted a discrepancy between the identified need for a CGA and actual referral rates to geriatric services from treating physicians. Future work is needed to understand the contextual factors influencing the ability to identify high-risk older ED patients who may benefit from a CGA and those factors that inhibit referral from the ED.
